# Fluid Overload Phenotypes in Critical Illness—A Machine Learning Approach

**DOI:** 10.3390/jcm11020336

**Published:** 2022-01-11

**Authors:** Anna S. Messmer, Michel Moser, Patrick Zuercher, Joerg C. Schefold, Martin Müller, Carmen A. Pfortmueller

**Affiliations:** 1 Department of Intensive Care Medicine, Inselspital, Bern University Hospital, University of Bern, 3010 Bern, Switzerland; michel.moser@insel.ch (M.M.); Patrick.zuercher@insel.ch (P.Z.); joerg.schefold@insel.ch (J.C.S.); carmen.pfortmueller@insel.ch (C.A.P.); 2 Department of Emergency Medicine, Inselspital, Bern University Hospital, University of Bern, 3010 Bern, Switzerland; martin.mueller2@insel.ch

**Keywords:** fluid resuscitation, fluid overload, intensive care, risk factors

## Abstract

Background: The detrimental impact of fluid overload (FO) on intensive care unit (ICU) morbidity and mortality is well known. However, research to identify subgroups of patients particularly prone to fluid overload is scarce. The aim of this cohort study was to derive “FO phenotypes” in the critically ill by using machine learning techniques. Methods: Retrospective single center study including adult intensive care patients with a length of stay of ≥3 days and sufficient data to compute FO. Data was analyzed by multivariable logistic regression, fast and frugal trees (FFT), classification decision trees (DT), and a random forest (RF) model. Results: Out of 1772 included patients, 387 (21.8%) met the FO definition. The random forest model had the highest area under the curve (AUC) (0.84, 95% CI 0.79–0.86), followed by multivariable logistic regression (0.81, 95% CI 0.77–0.86), FFT (0.75, 95% CI 0.69–0.79) and DT (0.73, 95% CI 0.68–0.78) to predict FO. The most important predictors identified in all models were lactate and bicarbonate at admission and postsurgical ICU admission. Sepsis/septic shock was identified as a risk factor in the MV and RF analysis. Conclusion: The FO phenotypes consist of patients admitted after surgery or with sepsis/septic shock with high lactate and low bicarbonate.

## 1. Introduction

Intravenous fluids are one of the most commonly applied therapies in the intensive care unit (ICU), and therefore, it is not surprising that optimizing this therapy is an ongoing issue in the management of the critically ill [1,2]. However, neither the ideal fluid nor fluid administration strategy has been found yet. One key limitation of currently available intravenous fluids is their transient effect on blood pressure, cardiac output, and peripheral perfusion due to third space extravasation through capillary leakage [3,4,5,6]. A liberal approach to fluid administration in critical illness thus often results in significant fluid overload (FO) in critically ill patients [7]. The association between FO, or a positive fluid balance, and mortality in critically ill has been shown in several studies [8,9,10,11,12]. Further, FO has an impact on other important outcomes, such as increased risk of acute kidney failure [10,13] and need for mechanical ventilation [14,15].

While awareness for the detrimental effects of FO in the critically ill has risen considerably during the last decade [12], and strategies to minimize FO were developed [16,17,18] and are currently under investigation [19], less effort has been undertaken to investigate factors that lead to FO in the critically ill. However, such an analysis is crucial to gain further insights on how FO in the critically ill can be minimized. Further, adult ICU patients are an extremely heterogenic group of patients and current trends in critical care research go towards characterizing “phenotypes” of critically ill patients [20,21,22,23]. Daulasim et al. recently discussed the importance of hemodynamic phenotypes to individualize the management of patients with septic shock [23]. Identifying a subgroup of patients especially particularly prone to FO in intensive care could be an essential step to optimize fluid management in the critically ill. Therefore, the aim of this retrospective cohort study is to identify factors contributing to FO in the critically ill and derive “FO phenotypes” by using machine learning techniques.

## 2. Materials and Methods

### 2.1. Setting & Study Design

This single-center retrospective cohort study was conducted at the Inselspital, University Hospital of Bern, Switzerland. Our unit consists of a large mixed 65-bed ICU and intermediate care unit (IMC) with board certified Intensive Care specialist in charge 24 h/7 d. We used patient record data to investigate factors associated with FO in adults admitted to our ICU from 1 January 2014 to 30 June 2018. The study was approved by the competent ethics committee of the Canton of Bern, Switzerland (Kantonale Ethikkommission Bern, EC no.: 2018-00436) and individual informed consent was waived by the ethics committee. The study was conducted in accordance with the Declaration of Helsinki.

**Figure 1:**
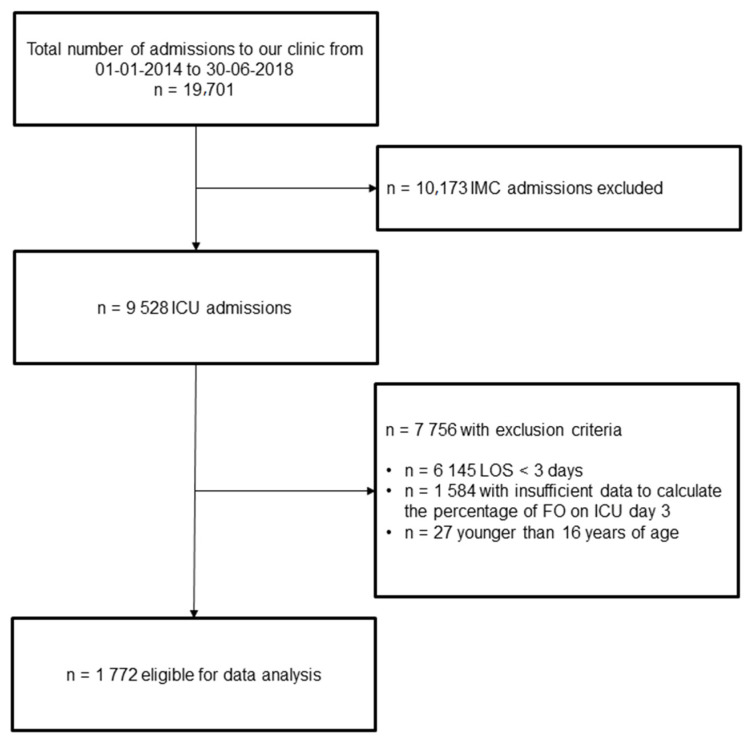
STROBE Flow chart. FO: fluid overload; ICU: intensive care unit; IMC: intermediate care unit; LOS: length of stay.

We included all adult ICU patients admitted during the study period with an ICU stay of at least three days. Exclusion criteria were as follows: (i) patients younger <16 years, (ii) insufficient data to calculate the percentage of FO (missing body weight, fluid input, or fluid output data). See Figure 1 for the STROBE (Strengthening the Reporting of Observational studies in Epidemiology) flowchart.

### 2.2. Data Collection & Extraction

This project is part of a large database on fluids, FO, and electrolyte disorders in the critically ill. Data for this project was provided by the Insel Data Coordination Lab (IDCL) and was extracted from our hospital’s electronic medical databases (SAP ERP 6.07/Inselspital Bern © SAP Schweiz 2018, Centricity Critical Care 8.1 © GE Electric Company, Boston, MA, USA, 2018, Xserv.4 R19.3 © ixmid GmbH, Köln, Germany, 2020, ipdos V7.16, © CompuGroup Medical Schweiz AG, Bern, Switzerland).

Eligible patients were identified through search in the hospitals administrative electronic database (SAP). We extracted the following variables on patients included: demographic data (e.g., age, sex), diagnosis and comorbidities, admission data including body weight, reason(s) for admission, and need for mechanical ventilation or vasopressors, as well as laboratory findings at ICU admission (defined as baseline). The percentage of FO on ICU day three was calculated (see definition below). Diagnosis and underlying diseases were based on International Statistical Classification of Diseases and Related Health Problems, 10th revision (ICD-10). Mortality data was extracted from the Swiss National Death Registry (ZAS, Zentrales Sterberegister).

### 2.3. Statistical Analysis

The statistical analysis was performed in Stata 16.1 (StataCorp, College Station, TX, USA) and R (V4.1.0, PBC, Boston, MA, USA; http://www.rstudio.com (accessed on 18 May 2021)).

Potential predictor variables were compared between the status of FO on day three (binary, >5% vs. ≤5%) using Chi-square tests (categorical variables) and the Wilcoxon rank sum test (continuous variables). Univariable logistic regressions with FO on day three as the outcome and all potential predictors as exposure were performed with the odds ratio (OR) accompanied by 95% confidence interval (CI) as the effect size. Five explanatory variables contained missing values (Acute Physiology And Chronic Health Evaluation (APACHE) IV score at admission, bicarbonate, sodium, lactate, and creatinine) and were imputed using multiple imputation (no. datasets = 10, see also Supplementary File, Figure S1) with Predictive Mean Matching as implemented in R package mice (mice v3.13.0., [24]). Each case with missing data was matched to the 10 cases having the closest predicted values (k = 10).

For further analysis the primary dataset was split into a training (70%) and validation set (30%) to allow evaluation of the performance of different models.

All variables of the univariable analysis that showed a significant association with FO (*p*-value < 0.05) were selected and used for final inference of the binary outcome of FO at day three. To compare and choose different approaches describing the relationship of the found explanatory variables on FO at day three, methods were applied to the imputed data sets, namely (i) logistic regression, (ii) random forest (randomForest 4.6-14, [25]), (iii) fast frugal trees [26], and classification decision trees [27]. All models are described in more detail with literature suggestions in Table S1 (Supplemental File). For each method, final model performance estimates were retrieved by pooling results from the 10 imputed data sets. Area under the curve (AUC) and the receiver operating characteristic curve were computed on the independent validation dataset and used to compare model performances. The DeLong test [28] was used to test for significant difference between the AUC of different models. As opposed to tree-based models, logistic regression models become unstable in the presence of multicollinearity, therefore, variance inflation factor was computed for explanatory variables and variables excluded if they exceeded a score of two [29]. For illustration and interpretation, single decision trees and fast frugal trees were trained on imputed dataset No. 1 and trees with maximized balanced accuracy (average of sensitivity and specificity) were reported. In addition to significance of logistic regression coefficients, importance of explanatory variables in predicting FO was evaluated using random forest feature importance and Boruta feature importance [30].

### 2.4. Definitions

#### 2.4.1. Fluid Overload

Generally, it must be noted that fluid overload (excess fluid) and a positive fluid balance is not the same and should be separated carefully. Clinically, fluid overload usually implies a degree of fluid accumulation into the tissues (e.g., peripheral edema or pulmonary edema), while a positive fluid balance simply reflects that fluid input is greater than fluid output. Almost all patients suffer from some degree of fluid accumulation while in the ICU (=have a positive fluid balance), and a positive fluid balance does not per se imply that the patient is fluid overloaded, therefore, the term “positive fluid balance” is a misleading surrogate marker for FO. The important question is beyond what threshold fluid accumulation becomes harmful for critically ill patients [12,31]. It was widely shown that accumulation of fluids of more than 5% of bodyweight results in a significant increase in mortality and morbidity for critically ill patients.

Thus, this weight-based definition (increase in body weight after admission of >5%) is considered the most accurate definition for fluid overload in critical care, and it is also widely used in nephrological research [32,33,34,35,36,37,38,39,40,41]. We thus have used this definition for our work. FO was estimated as the total fluid balance relative to the baseline body weight (percent FO) using following formula [32,33,42]: (cumulative fluid intake − cumulative fluid losses)/(admission weight) × 100. A patient being fluid overloaded was defined as a FO of five percent or more [42].

#### 2.4.2. Cumulative Fluid Intake and Losses

Cumulative fluid intake accounts for all fluids a patient may have received including fluids with nutrition, fluids for resuscitation, baseline fluids, fluid with medication, and oral fluids (e.g., water, coffee, soft drinks), as well as blood products (e.g., red blood cells, fresh frozen plasma). Cumulative fluid losses include urinary losses, ultrafiltration in case of dialysis, all drainage losses, fecal losses, and evaporation. Evaporation is an estimate based on the patients age, body surface area, and body temperature (fever correction).

## 3. Results

A total of 1772 patients suitable for analysis were identified (see Figure 1). The median age was 63 years, and 1211 patients were male (68.3%). Among the cohort, 387 (21.8%) of the patients fulfilled the criteria for FO at day three. The median percentage of FO in the FO group was 8.6% vs. −0.4%. Cumulative fluid intake was 12,644 mL in the FO group vs. 5976 mL in the non-FO group; *p* < 0.01. Total fluid losses amounted to 5749 mL in the FO group vs. 6603 mL in the non-FO group; *p* < 0.01. Patients in this group were older (median age 66, vs. 62; *p* < 0.039) and significantly more often had a history of chronic liver (19.9% vs. 11.9%; *p* < 0.01) or chronic kidney disease (39.9% vs. 27.3%; *p* < 0.001) when compared to patients without FO on day three after admission to the ICU (see also Supplemental Table S2 for baseline characteristics).

### 3.1. Univariable and Multivariable Analysis

The results of the univariable and multivariable analyses are depicted in Table 1. The condition with the highest odds ratio (OR) for FO was surgery prior to ICU admission (OR 4.20), followed by admission status (planned admission OR 3.11), a history of organ transplantation (OR 2.93), and sepsis and septic shock as reasons for ICU admission (OR 1.91). In the MV, baseline lactate (OR 1.28), surgery prior to ICU admission (OR 2.35), diagnosis of septic shock (OR 2.05), need for mechanical ventilation at ICU admission (OR 1.56), and planned ICU admission (OR 1.70) were identified as independent predictors of FO on day three. High bicarbonate baseline levels (OR 0.89), non-traumatic neurological disease (OR 0.33), and male sex (OR 0.71) are inversely associated with the development of FO on day thee after adjustment. Creatinine at baseline and APACHE IV as markers of disease severity had no impact on the development of FO on day three. See also Table 1.

### 3.2. Fast and Frugal Tree

**Figure 2:**
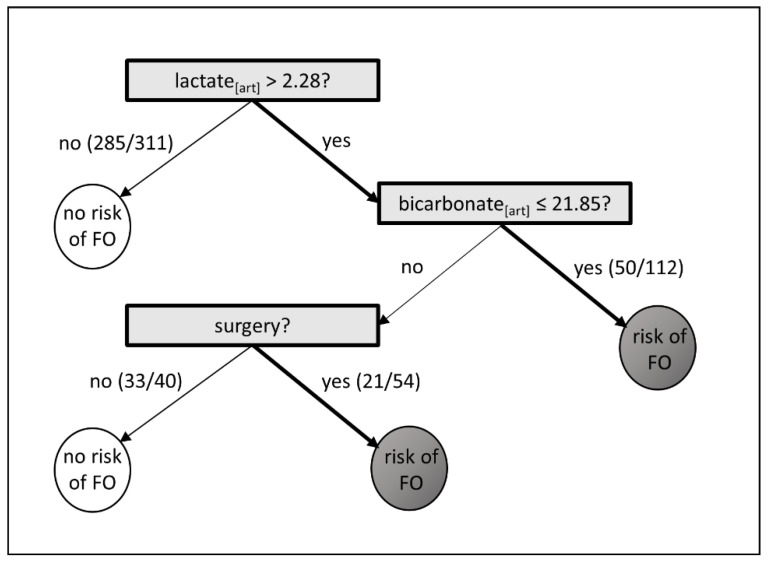
Fast and Frugal Tree. Variables are considered sequentially, with possible stop decision after each question. White circles indicate no risk of fluid overload (FO), and dark grey circle indicate a risk of FO Lactate and bicarbonate were measured in arterial blood samples (art).

The fast and frugal tree (FFT) analyses in 517 patients determined a pathway starting with baseline lactate, followed by surgery prior to admission and baseline bicarbonate levels. Lactate levels > 2.28 mmol/L, bicarbonate levels ≤ 21.85 mmol/L, and surgery prior to ICU admission correctly decided for FO in 68% (71/104) patients, and for no FO in 76% (318/413), see Figure 2. Overall, the sensitivity was 68.3% and specificity 77.0%.

### 3.3. Classification Decision Tree

**Figure 3:**
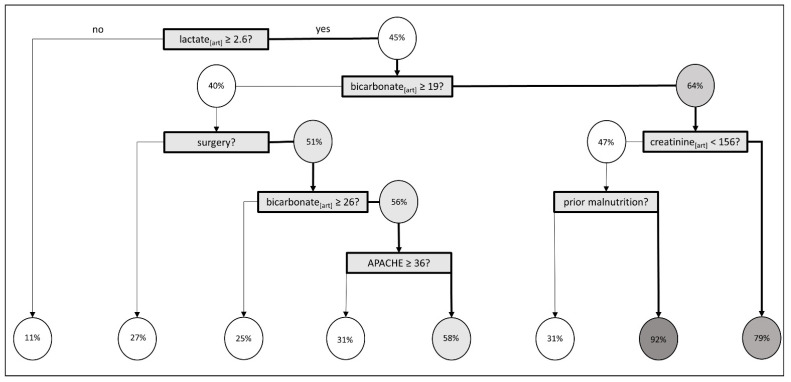
Classification Decision Tree. Percentages indicate the proportion of patients with fluid overload (FO). The darker the grey, the higher the percentage of patients developing a FO. Acute Physiology and Chronic Health Evaluation (APACHE) IV. Lactate and bicarbonate were measured in arterial blood samples [art].

The classification decision tree revealed lactate ≥2.6 mmol/L to be the most important predictor for FO at ICU day three followed by bicarbonate <19.0 mmol/L. Kidney function at ICU admission (baseline creatinine > 156 μmol/L) plays a role in the third generation and APACHE IV of ≥36 in the 5th generation (see Figure 3). Sensitivity was 89.1% and specificity was 45.2%.

### 3.4. Random Forest and Boruta Importance

After application of the Boruta algorithm, 13 variables were significantly associated with FO at day three. The highest importance to predict FO at day three had lactate and bicarbonate levels at admission and surgery prior to admission (Boruta importance 33.10, 20.15, respectively 12.50). In Table 2, we summarized the variables from high importance to low importance, a visual distribution of variable importance is provided in the Supplementary File, Figure S2.

### 3.5. Comparison of Statistical Models

**Figure 4:**
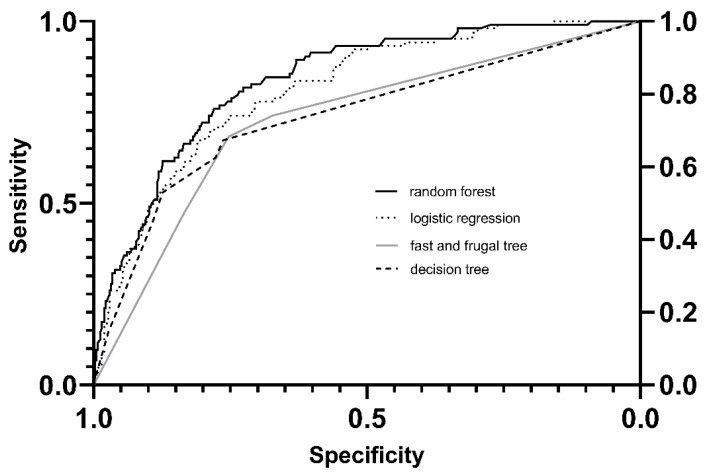
Comparison of Models.

Comparing the random forest model, the fast and frugal tree, the classification decision tree, and the logistic regression (see Figure 4), the best AUC for predicting FO on day three in critically ill patients was the random forest model with 0.84 (95% CI 0.79–0.86). The logistic regression had an AUC of 0.81 (95% CI 0.77–0.86), followed by an FFT of 0.74 (95% CI 0.69–0.79), and a DT AUC of 0.73 (95% CI 0.68–0.78). While the AUC of the logistic regression and the random forest model did not differ significantly (*p* < 0.251), the AUC of the fast frugal trees and the classification decision tree was significantly lower than that of the logistic regression model and the random forest model (all *p* < 0.0001). The AUC of the FFT and the DT did not differ significantly (*p* < 0.72).

## 4. Discussion

This analysis compromising four different approaches, including machine learning techniques, revealed that patients admitted with high lactate and low bicarbonate with sepsis/septic shock and those admitted after surgery to be at increased risk to suffer from FO at ICU day three (the FO phenotypes). Disease severity and renal factors (acute or chronic) seem to be less important contributors.

Our analysis identifies high lactate to be a major determinate for FO at ICU day three well reflect current clinical practice, as lactate has traditionally been used to guide fluid resuscitation therapy in critically ill patients [43,44,45].

The concept behind lactate-guided fluid administration is mainly based on the idea that increased lactate levels in the critically ill may reflect cellular dysoxia and thus inadequate tissue perfusion [46,47]. Several investigations identified elevated lactate values to be independently associated with ICU mortality [48,49,50] and early lactate clearance to be beneficial for ICU outcomes [45,51]. The LACTATE study revealed that lactate-guided therapy significantly reduces hospital mortality and several important endpoints when adjusted for predefined risk factors [45]. Thus, guidelines and consensus statements, including surviving sepsis guidelines, were proposed to achieve a reduction in serum lactate by administration of crystalloids (i.e., 30 mL/kg for initial resuscitation, followed by additional fluid if necessary) [43,44].

Controversially, in the recently published ANDROMEDA-SHOCK trial, lactate-guided resuscitation of patients with septic shock did not result in less mortality than perfusion guided treatments (28-day mortality 43.4% versus 34.9%) [52]. Although the ANDROMEDA-SHOCK trial missed the mark for statistical significance (*p* < 0.06), a post-hoc analysis of the same trial using the Bayesian approach revealed a posterior median odds ratio for 28-day mortality of 0.61 [53].

Thus, it may be argued that lactate-guided resuscitation might result in increased mortality in the critically ill. The reason might be the increased amounts of fluids administered when this resuscitation strategy is used (e.g., ANDROMEDA-SHOCK trial total fluid balance, 2767 mL (SD 1749 mL) in the lactate guided arm, 2359 mL (SD 1344 mL) in the tissue perfusion arm; *p* < 0.01) [45,52].

A crucial question remains; what lactate level is clinically important and warrants treatment? A prospective observational study evaluated the use of lactate as a prognostic marker in patients with suspected infection after controlling for hemodynamic status and co-morbidities [49]. This study reveals that the adjusted OR for mortality in patients with lactate levels of 2.5–3.9 mmol/L was 2.2 (95% CI 1.1–4.2) while it increased to 7.1 (95% CI 3.6–13.9) for patients with a lactate level > 4.0 mmol/L [49]. This implies that occult hypoperfusion (i.e., not related to shock state) results in excess mortality above 4.0 mmol/L and stands in contrast to the proposed lactate threshold of >2 mmol/L [43]. A cut-off of 4.0 mmol/L to guide fluid resuscitation in the critically ill seemed safe and feasible in the critically ill in the first analysis [18] and is used in several trials on fluid restriction or de-resuscitation that are currently running [19,54]. As our study shows, lactate seems to be a major determinant of fluid administration and FO. Therefore, increasing the lactate threshold for fluid resuscitation—provided it proves to be safe in the currently running trials—may help to reduce FO in the future.

In addition, our study reveals that low bicarbonate—a marker of metabolic acidosis [55]—is associated with FO. This is not surprising, as it is often combined with elevated lactate levels, which has shown to be an associated factor of FO in the critically ill. However, low bicarbonate or metabolic acidosis has multiple etiologies [55,56], and in general fluid resuscitation (except in form of bicarbonate replacement for severe acidosis) is not recommended for the management of metabolic acidosis [57,58]. In addition, low bicarbonate, in association with FO, could also be a reverse association as excessive fluid administration can also lead to metabolic acidosis with a decrease in bicarbonate levels if, for example, 0.9% saline is used [59,60,61]. This study revealed that patients after surgery, as well as patients with sepsis/septic shock, are especially prone to FO. As discussed above, sepsis can be associated with elevated lactate and the surviving sepsis guideline [62,63] recommends the administration of a minimum of 30 mL/kgBW crystalloid fluid within the first hour of treatment with the aim of achieving lactate clearance and stabilizing hemodynamics. However, this “strong” recommendation is not based on solid evidence [62]. Sepsis/septic shock is not, per se, a volume depleted state, it is the microcirculatory alterations combined with vasodilatation and cardiac dysfunction that lead to a reduction in stressed volume and cardiac output [64]. Thus, the purpose of fluid resuscitation in patients with sepsis is to increase stressed volume and mean systemic filling pressure, thus increasing cardiac preload via increased gradient for venous return [64]. The same applies in part to patients undergoing surgery, where, in addition to surgery associated fluid loss, narcotic agents cause vasodilatation and cardiac depression. However, only half of the patients with sepsis/septic shock or even less during surgery are fluid responsive and thus benefit from fluid administration [65,66,67]. Nevertheless, large amounts of fluids are administered to these patient groups leading to FO, as our study shows.

In addition, both sepsis/septic shock and surgery are also associated with capillary leakage due to glycocalyx breakdown caused by circulating inflammatory mediators [68]. Recent data suggest that this effect might even be promoted by intravenous fluid administration [68] through amplifying endothelial dysfunction. These iatrogenic injuries might explain the results of two randomized trials showing that early aggressive fluid boluses in sepsis worsened survival [69,70]. As our study shows, the evaluation of strategies to minimize FO (e.g., restrictive fluid strategies or de-resuscitation protocols) in patients with sepsis/septic shock and surgery is highly warranted. Several investigations [19,58,71] are currently running and will potentially shed further light as to whether fluid restriction or de-resuscitation may improve FO and outcome in the critically ill.

### Limitations

This study has several limitations that warrant discussion. First, this is a single-center study; hence, external validity is yet to be proven. Second, as this is a retrospective evaluation, some of the data were incomplete and had to be imputed. Although we performed ten imputations with good reproducibility (see Supplementary File, Figure S1), there is still the potential of bias. In addition, we adjusted our analysis for confounders (i.e., disease severity, age, and sex). However, due to the retrospective design we cannot exclude a potential reverse causality regarding disease severity. It may be possible, even though this is not reflected by our findings concerning APACHE IV score, that patients with elevated lactate and metabolic acidosis are sicker, tend to have higher fluid retention, and thus are more prone to FO.

Third, while the bodyweight used for calculation of FO was retrieved from medical records or from the patient or his/her relatives if possible, it was estimated at ICU admission by the treatment team for cases where the information could not be found elsewhere, this creates a potential for bias. Fourth, even though a considerable body of evidence showed fluid accumulation beyond 5% of body weight to be harmful for critically ill patients, this definition serves as a surrogate for FO in the critically ill only. Importantly, it does not include clinical signs such as edema formation or pleural effusion and, therefore, might be a source of bias. Fifth, we used creatinine at admission as a surrogate for acute kidney injury (AKI), which might not be reliable. However, as baseline creatinine was not known for our patients, and the retrospective calculation of baseline creatinine was not reliable [71,72,73], we settled for creatinine at admission as a surrogate for AKI in the knowledge of this limitation. Last, our study has a retrospective design.

## 5. Conclusions

This study reveals that the FO phenotypes consist of (I) patients admitted with sepsis/septic shock with a high lactate and a low bicarbonate and (II) patients after surgery with the same laboratory features. This study highlights the importance of diagnosis and laboratory markers as early as ICU admission to identify patients at risk for FO. Most interestingly, renal factors and disease severity at admission do not seem to significantly influence the risk of developing FO during an ICU stay. In the future, tailored fluid minimization strategies for patients admitted with sepsis/septic shock and surgery should be investigated.

## Figures and Tables

**Table 1:** Univariable and Multivariable Analysis for Fluid Overload at Day 3.

	Univariable Model			Multivariable Model		
Variable	Odds Ratio	95% CI	*p*-Value	Odds Ratio	95% CI	*p*-Value
***Demographics***						
Age	1.01	(1.00–1.01)	0.081			
Sex (male)	0.74	(0.58–0.93)	**0.012**	0.71	(0.50–0.99)	**0.046**
APACHE IV	1.03	(1.02–1.04)	**<0.001**	1.00	(0.99–1.02)	0.637
Admission type (planned)	3.11	(2.35–4.11)	**<0.001**	1.70	(1.07–2.70)	**0.024**
***Past Medical History***						
Immune deficiency	1.48	(1.09–2.02)	**0.012**	1.02	(0.59–1.76)	0.938
Chronic kidney disease	1.72	(1.36–2.18)	**<0.001**	1.05	(0.74–1.49)	0.795
Chronic liver disease	1.84	(1.36–2.47)	**<0.001**	1.55	(0.98–2.44)	0.061
Cancer	1.00	(0.69–1.46)	0.981			
Organ transplantation	2.93	(1.81–4.75)	**<0.001**	1.23	(0.52–2.89)	0.634
Arterial hypertension	1.09	(0.87–1.37)	0.465			
Diabetes mellitus (any type)	1.06	(0.64–1.75)	0.814			
Malnutrition	1.72	(1.33–2.23)	**<0.001**	1.08	(0.74–1.58)	0.697
***Diagnosis at ICU admission***						
Sepsis/septic shock	1.91	(1.51–2.42)	**<0.001**	2.05	(1.44–2.91)	**0.007**
Respiratory failure	0.92	(0.72–1.17)	0.483			
Heart failure and cardiogenic shock	1.70	(1.34–2.16)	**<0.001**	0.96	(0.67–1.37)	0.827
Pancreatitis	1.06	(0.48–2.36)	0.883			
Major trauma	0.62	(0.44–0.88)	**0.008**	1.02	(0.61–1.71)	0.927
Non-traumatic neurological disease	0.16	(0.09–0.28)	**<0.001**	0.33	(0.16–0.71)	**0.005**
Surgery prior admission	4.20	(3.30–5.35)	**<0.001**	2.35	(1.52–3.62)	**<0.001**
Infection (any type) at admission	0.85	(0.67–1.08)	0.176			
***Treatment at ICU admission***						
Mechanical ventilation	2.31	(1.84–2.91)	**<0.001**	1.56	(1.10–2.20)	0.012
Vasoactives	1.60	(0.98–2.62)	0.062			
***Lab values at admission***						
Sodium (mmol/L)	0.97	(0.95–0.99)	**0.012**	0.97	(0.94–1.01)	0.103
Bicarbonate (mmol/L)	0.85	(0.83–0.88)	**<0.001**	0.89	(0.85–0.93)	**<0.001**
Lactate (mmol/L)	1.50	(1.42–1.60)	**<0.001**	1.28	(1.18–1.39)	**<0.001**
Creatinine (μmol/L)	1.00	(1.00–1.00)	**<0.001**	1.00	(1.00–1.00)	0.633

Acute Physiology and Chronic Health Evaluation (APACHE), Intensive Care Unit (ICU), Confidence Interval (CI). Bold numbers indicate significant *p*-values.

**Table 2:** Important Variable Selection with Boruta Algorithm.

Variable	Mean Imp	Median Imp	Min Imp	Max Imp	Norm Hits	Decision
Lactate (mmol/L)	32.77	32.68	29.04	35.91	1	Confirmed
Bicarbonate (mmol/L)	19.92	20.04	16.59	23.41	1	Confirmed
Surgery prior to admission	12.3	12.21	9.24	15.16	1	Confirmed
Sepsis/septic shock	6.79	6.64	4.79	10.19	1	Confirmed
Creatinine (μmol/L)	6.28	6.24	3.51	8.32	1	Confirmed
Non-traumatic neurological disease	5.77	5.79	2.76	7.79	1	Confirmed
Chronic liver disease	5.05	5.19	2.27	8.23	0.93	Confirmed
Admission type (planned)	4.78	4.79	1.37	7.37	0.95	Confirmed
Sodium (mmol/L)	4.18	4.19	2.17	7.02	0.86	Confirmed
APACHE IV	3.97	3.95	1.16	7	0.87	Confirmed
Chronic kidney failure	3.46	3.35	0	6.2	0.76	Confirmed
Hx of organ transplantation	3.22	3.22	0.37	5.58	0.71	Confirmed
Mechanical ventilation (at admission)	3.14	3.23	−0.68	5.15	0.7	Confirmed
Hx of malnutrition	2.9	2.96	−0.2	5.41	0.68	Confirmed
Heart failure/cardiogenic shock	2.54	2.69	−0.3	4.36	0.54	Tentative
Sex (male)	1.41	1.21	−0.93	3.38	0.04	Rejected
Hx of immune deficiency	−0.03	−0.13	−3.17	2.26	0.01	Rejected
Major trauma	−0.13	−0.49	−1.46	1.54	0	Rejected

Variable selection for contribution to FO on day three after ICU admission. History of (Hx). The laboratory markers lactate, sodium, bicarbonate, creatinine, and the APACHE IV score were measured at admission (=baseline). Mean Imp—the mean of IMp, Median Imp—the median of IMp, Min Imp—the minimum of IMp, Max Imp—the maximum of IMp, Norm Hits-the number of hits normalized to number of importance source runs, where. IMp is the importance measure computed over multiple iterations.

## Data Availability

The data is available on request from the author.

## Supplementary Materials

The following are available online at https://www.mdpi.com/article/10.3390/jcm11020336/s1, Figure S1. Imputed Variables, Figure S2. Boruta Variable Importance, Table S1: Machine Learning Methods Used, Table S2: Baseline characteristics. References [25,26,74,75,76,77,78,79] are cited in the Supplementary Materials.
