# Supplementary material for: Fluid Overload Phenotypes in Critical Illness—A Machine Learning Approach
Source: J Clin Med. 2022 Jan 11;11(2):336. doi: 10.3390/jcm11020336 (PMC8780174; doi:10.3390/jcm11020336)
Supplement: Supplementary file 1 [file jcm-11-00336-s001.zip › jcm-1513092-supplementary.pdf]

Journal of
Clinical Medicine

MDPI

Supplementary File

This appendix has been provided by the authors to provide additional information

Supplement to: Messmer AS, Moser M, Zuercher P, Schefold JC, Müller M, Pfortmueller CA. **Fluid overload phenotypes in critical illness – a machine learning approach**

Corresponding author
Anna Messmer, MD
Department of Intensive Care,
Inselspital, Bern University Hospital and University of Bern
Freiburgstrasse 10
3010 Bern
Switzerland
ORCID: 0000-0002-3206-9112

Content

Supplemental Table S1. Machine Learning Methods Used 3
Supplemental Table S2. Baseline characteristics 4
Supplemental Figure S1. Imputed Variables 5
Supplemental Figure S2. Boruta Variable Importance 6
References 7

Supplemental Table S1. Machine Learning Methods Used

Method	Description	Suggested Literature for further reading
Tree-based models	All here applied machine-learning methods stem from tree-based models which share a tree-like layout applied as a set of ordered, conditional rules in the form "If A, then B, else …" that are applied to cues (clinical features, variables) sequentially until a decision is reached. They are non-linear models and trained on data where the outcome is known (supervised learning).	Handelman et al., 2018 [1]<br>Banerjee et al., 2019 [2]
Decision trees	Most physicians will be familiar with decision trees, presented by branching decisions based on patient data. Generally, a decision tree is comprised of a sequence of nodes, representing cue-based questions, branches, representing answers to those questions, and leaves, representing decisions. These trees are generated by reviewing the evidence and making suggestions based on clinical data and physician opinion. With increasing data load and complexity of analysis, decision trees can be constructed by training decision tree algorithms on large databases of patient cases to construct a decision tree based on variables which can determine the highest separability on the desired categories. This allows for the partition of observations in a hierarchical basis using a set of simple decisions and allows for feature selection of discriminant variables.	Handelman et al., 2018 [1]<br>Philips et al., 2017 [3]<br>Kotsiantis, 2013 [4]
Fast and frugal trees	Fast-and-frugal trees are decision trees with strict restrictions on the size and shape to solve binary classification tasks while keeping the tree simple and easy to interpret. Cues (clinical variables) are sequentially ordered, where at every branching point exactly two extending branches are allowed and either one or both branches is an exit branch leading to a final decision. This allows the algorithm to operate speedily, with little information and makes decisions to be optimized with as few cues as possible.	Martignon et al., 2003 [5]
Random forests	Single decision trees use only a partial subset of all cue information available since, once a decision is made no additional information of this cue is considered for decisions to follow. Random forests presents an alternative to this restrictions by generating collections of decision trees and aggregating their decisions into one final result. By training individual trees on different samples of the data, random forests are able to limit overfitting without substantially increasing error due to bias which makes them very powerful models. This comes with the cost of reduced interpretability, as gaining insight on prediction rules is hard due to the large number of trees.	Breiman, 2001 [6]<br>Couronné et al., 2018 [7]<br>Liaw and Wiener, 2002 [8]

Supplemental Table S2. Baseline characteristics

Variable	All	Fluid overload at day 3		
		yes	no	
	n = 1772	n=387	n=1385	*p-value*
Demographics[1]				
Age	63 [51 - 73]	66 [52 - 74]	62 [51 - 72]	**0.039**
Sex (male)	1211 (68.3)	244 (63.0)	967 (69.8)	**0.011**
APACHE IV	21 [5 - 29]	26 [14 - 33]	19 [4 - 28]	**<0.001**
LOS (days)	17 [10 - 28]	21 [14 - 36]	16 [9 - 26]	**<0.001**
Past Medial History[2]				
Immune deficiency	242 (13.7)	68 (17.6)	174 (12.6)	**0.011**
Chronic kidney disease	530 (29.9)	152 (39.3)	378 (27.3)	**<0.001**
Chronic liver disease	242 (13.7)	77 (19.9)	165 (11.9)	**<0.001**
Cancer	178 (10.0)	39 (10.1)	139 (10.0)	0.981
Organ transplantation	71 (4.0)	31 (8.0)	40 (2.9)	**<0.001**
Arterial hypertension	736 (41.5)	167 (43.2)	569 (41.1)	0.465
Diabetes mellitus (any type)	92 (5.2)	21 (5.4)	71 (5.1)	0.814
Malnutrition	366 (20.7)	109 (28.2)	257 (18.6)	**<0.001**
Diagnosis at ICU admission[2]				
Sepsis/septic shock	537 (30.3)	161 (41.6)	376 (27.1)	**<0.001**
Respiratory failure	571 (32.2)	119 (30.7)	452 (32.6)	0.483
Heart failure and cardiogenic shock	486 (27.4)	140 (36.2)	346 (25.0)	**<0.001**
Pancreatitis	35 (2.0)	8 (2.1)	27 (1.9)	0.883
Major trauma	263 (14.8)	41 (10.6)	222 (16.0)	**0.008**
Non-traumatic neurological disease	277 (15.6)	14 (3.6)	263 (19.0)	**<0.001**
Surgery prior to admission	433 (24.4)	185 (47.8)	248 (17.9)	**<0.001**
Infection (any type) at admission	689 (38.9)	139 (35.9)	550 (39.7)	0.176
Treatment at ICU admission[2]				
Mechanical Ventilation	790 (44.6)	235 (60.7)	555 (40.1)	**<0.0001**
Vasoactiva	79 (4.5)	24 (6.2)	55 (4.0)	0.060
Lab values at admission[1]				
Sodium (mmol/l)	137.8 [135.3 - 140.3]	137.0 [134.7 - 139.7]	138.0 [135.5 -140.5]	**<0.001**
Bicarbonate (mmol/l)	22.6 [20.3 - 25.0]	21.1 [18.3 - 23.1]	23.2 [21.0 - 25.7]	**<0.001**
Lactate (mmol/l)	2.0 [1.2 - 3.3]	3.5 [2.3 -5.4]	1.7 [1.1 -2.6]	**<0.001**
Creatinine (mmol/l)	99 [70.5 - 150.7]	121 [81.5 – 185.0]	95 [68.0 - 137.5]	**<0.001**

[1] median [IQR]; [2] n (%). *APACHE IV* = Acute Physiology And Chronic Health Evaluation*; LOS* = Length of Hospital Stay; *ICU* = Intensive Care Unit; *IQR* = Interquartile Range

Supplemental Figure S1. Imputed Variables

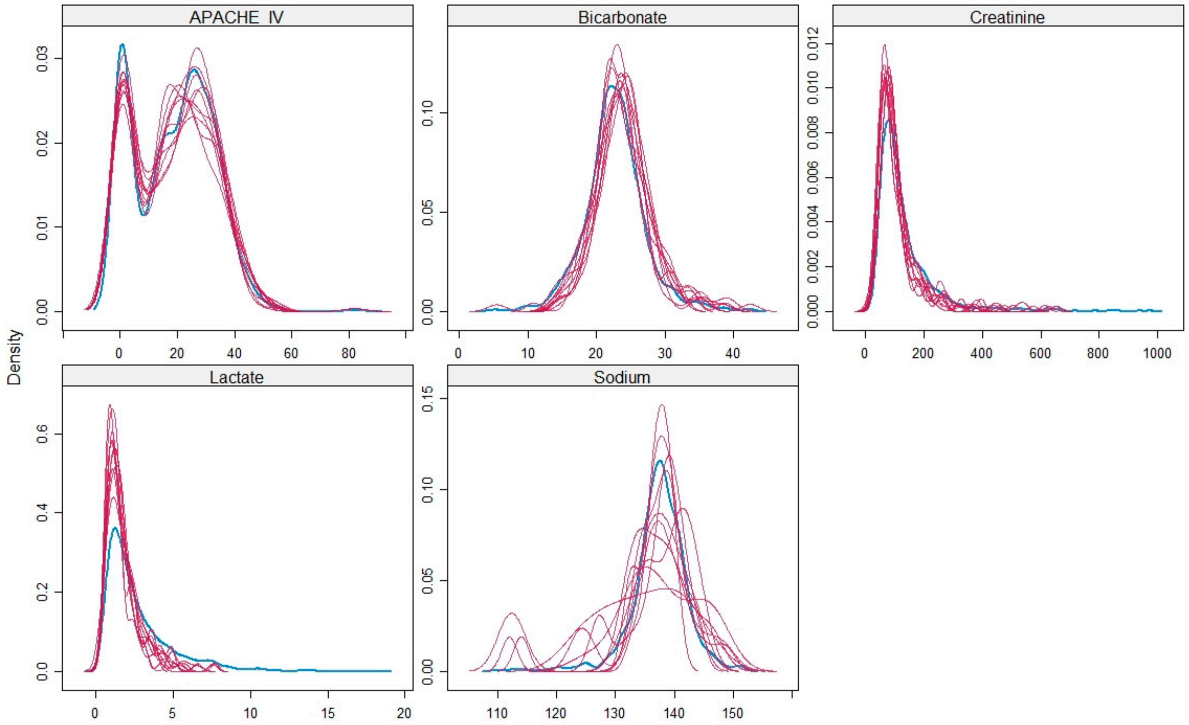

Explanatory variables on admission with missing values (%): APACHE IV score (24.2%), bicarbonate (6.6%), sodium (1.1%), lactate (6.0%), and creatinine (7.7%). Variables were imputed using multiple imputation (no. datasets = 10). The density of the imputed data for each imputed dataset is showed in magenta while the density of the observed data is showed in blue.

Supplemental Figure S2. Boruta Variable Importance

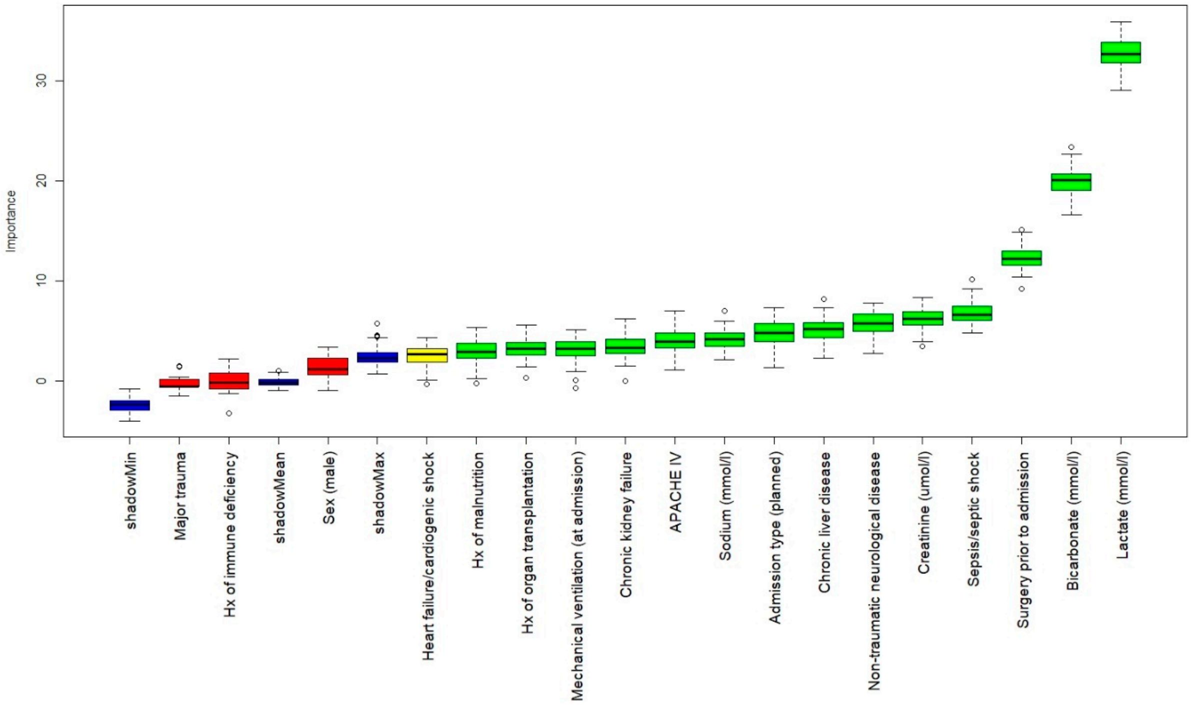

Variable selection for contribution to FO on day 3 after ICU admission. *Hx* = History of, *APACHE IV* = Acute Physiology And Chronic Health Evaluation, Green columns were confirmed as being "important", yellow columns represent "tentative" attributes, and red columns rejected variables.

References

1. Handelman, G.S.; Kok, H.K.; Chandra, R.V.; Razavi, A.H.; Lee, M.J.; Asadi, H. eDoctor: machine learning and the future of medicine. *J. Intern. Med.* **2018**, *284*, 603–619.
2. Banerjee, M.; Reynolds, E.; Andersson, H.B.; Nallamothu, B.K. Tree-Based Analysis. *Circ. Cardiovasc. Qual. Outcomes*. **2019**, *12*, e004879.
3. Phillips, N.D.; Neth, H.; Woike, J.K.; Gaissmaier, W. FFTrees: A toolbox to create, visualize, and evaluate fast-and-frugal decision trees. *Judgm. Decis. Mak.* **2017**, *12*, 344–368.
4. Kotsiantis S.B. Decision trees: a recent overview. *Artif. Intell. Rev*. **2013**, *39*, 261–283.
5. Martignon, L.; Vitouch, O.; Takezawa, M.; Forster, M.R. Naive and Yet Enlightened: From Natural Frequencies to Fast and Frugal Decision Trees. In *Thinking: Psychological Perspectives on Reasoning, Judgment and Decision Making*; Hardman, D., Macchi, L., Eds.; Wiley & Sons: New York, NY, USA, 2003, pp. 189–211.
6. Breiman, L. Random Forests. *Mach. Learn*. **2001**, *45*, 5–32.
7. Couronné R.; Probst, P.; Boulesteix, A.L. Random forest versus logistic regression: a large-scale benchmark experiment. BMC Bioinform. **2018**, *19*, 270.
8. Liaw, A.; Wiener, M. Classification and Regression by randomForest. *R News* **2002**, *2*, 18–22.
